# Surgical Management of Oro-Nasal Communication in Cocaine-Induced Lesions: Temporalis Muscle Flap with Le Fort I Osteotomy

**DOI:** 10.3390/jcm14062033

**Published:** 2025-03-17

**Authors:** Ettore Lupi, Alessandra Ciccozzi, Roberto Becelli, Mario Mannino, Sara Bernardi, Filippo Giovannetti

**Affiliations:** 1 Department of Biotechnological and Applied Clinical Sciences, University of L’Aquila, 67100 L’Aquila, Italy; 2 Department of Life, Health and Environmental Sciences, University of L’Aquila, 67100 L’Aquila, Italy; 3 Maxillofacial Unit, University “La Sapienza”, 00185 Rome, Italy

**Keywords:** temporal muscle, reconstructive surgery, maxillo mandibular reconstruction, health

## Abstract

**Background:** Cocaine is a recreational drug known for its negative impact on health and social and economic life. One of the complications for cocaine abusers is cocaine-induced midline destructive lesion (CIMDL) syndrome, which includes the extensive destruction of the osteocartilaginous structures of the nose, the sinus, and the palate. **Methods:** Here, we describe three cases of the surgical management of CIMDL using a temporalis muscle flap combined with a Le Fort I osteotomy, which allows sufficient space for the muscle to settle. **Results:** The addition of the osteotomy allowed better handling of the pedicled flap, with no signs of relapse in the reported cases and high patient satisfaction. **Conclusions:** CIMDL syndrome is an impairing disease that negatively affects the functionality of the nasal and oral cavities, as well as the aesthetic. Surgical repair using a pedicled flap is a valid option in rehabilitated and sober patients with endothelial damage.

## 1. Introduction

The widespread abuse of cocaine, particularly among young adults between ages 18 and 30, has resulted in many patients presenting with extensive facial destruction of the osteocartilaginous structures of the nose, the sinus, and the palate (cocaine-induced midline destructive lesion—CIMDL syndrome) [1].

Indeed, the most common way to absorb cocaine is via the nasal vascular supply, assuming it is ingested intranasally [2]. Repeated snorting leads to a cascade of ischemia, inflammation, micronecrosis, and infection of the nasal anatomical structures. This effect, together with sodium channel blockade (interfering with nerve transmission leading to an anesthetic effect and direct chemical damage from the adulterants with which the drug is “cut”), can lead to mechanical injury and necrosis of the mucosa and the surrounding osteocartilaginous structures, followed by macronecrosis up to perforation [3].

The pathogenesis of these lesions follows a vasculitis pattern that puts the diagnosis of CIMDL in a differential diagnosis with Wegner granulomatosis, granulomatosis with polyangiitis (GPA), sarcoidosis, or nasal non-Hodgkin’s lymphoma [4]. The diagnosis of CIMDL must include clinical and radiological parameters and at least two of the following conditions: nasal septal perforation, lateral nasal wall destruction, and hard palate involvement [5].

The various sinonasal structures are destroyed at different frequencies. Trimarchi et al. evaluated 25 patients with CIMDL, finding that the nasal septum was eroded in all patients [6]. The inferior, middle, and superior nasal turbinates were affected in 68%, 44%, and 16% of the patients, respectively. Palatal perforation was seen in 24% of the patients. Erosion of the lateral nasal walls was seen in only 20% of the patients [6].

In addition, cocaine impairs mucociliary transport, causing debris to accumulate [7]. Indeed, in healthy nasal tissue, the mucociliary system moves foreign particles to the nasopharynx, with a clearance time of approximately 20 min [8]. A cocaine concentration of 2.5% has been found to reduce clearance by 58%, and a concentration of 10% causes irreversible ciliary damage [7,8,9].

A range of midfacial destructive lesions have been documented, including perforation of the nasal septum, destruction of the lateral wall of the nasal cavities, and perforation with necrosis of the soft and hard palate. Giovannetti et al. reported mostly hard palate (77.7%) lesions [10].

All the inflammatory processes cause palatal defects, which create functional deficits in speech and swallowing [8]. As a result, patients typically complain of symptoms such as regurgitation of food and liquids in the nose, rhinolalia, anosmia, pain, and halitosis [6].

Hard palate defects can be treated with a prosthetic obturator or through local flaps, regional rotational flaps, and free microvascular flaps [4,7,8,9,10,11].

Regarding surgical reconstruction, several surgical techniques have been described, and according to the lesion’s extension, local mucoperiosteal flaps or tongue flaps (due to their intense vascularization) or nonpedicled flaps, usually forearm free flap approaches, can be chosen [9].

Chronic cocaine abuse repeatedly and constantly damages the vascular endothelium, not only locally in the midfacial structures but throughout the whole body. For example, the heart suffers at the ventricular level, with a decreased ejection fraction and increased end systole secondary to the interaction between cocaine and norepinephrine [12].

In addition, reports in the literature showed how chronic cocaine use impacts the nervous system through panhypopituitarism, dysfunction of the optic nerve, diffuse lesions in white matter, including the basal ganglia, proptosis, and visual dysfunction due to the destruction of the bony components of the orbital cavity [12].

In general, cocaine triggers the immune system and induces an inflammatory process, decreasing anti-inflammatory markers such as interleukin-10 and increasing pro-inflammatory cytokines (e.g., tumor necrosis factor-alpha and interleukin 1β). The activation of this cytokinin cascade contributes to vascular disease [12].

All of these considerations, combined with the addiction disturbance, make CIMDL rehabilitation a challenge.

The chronic abuse of cocaine causes an inflammatory process throughout the body. Although Colletti et al. underlined the success of free flaps [9], damage to facial vessels might compromise the long-term stability of the forearm flap. Pedicled local and regional flaps keep the vascular supply intact without needing microvascularization or other anastomoses.

If we look at other similar situations, where the bones combining the face’s midline possess a damaged vascular supply, such as oncologic sites, bone grafts have resulted in a lower failure rate than vascularized bone [13].

The patient should certainly refrain from cocaine consumption to prevent compromising surgical outcomes. Therefore, careful patient selection prior to interventions for oro-nasal fistula closure is fundamental.

Alternatively, prosthetic obturators can be an option to avoid the side effects and consequences of CIMDL [8]. Indeed, the obturator can be used when the disease is ongoing or if the patient is still using cocaine. The main disadvantages of this solution are follow-ups for adjustments and discomfort. Indeed, a good obturator should be stable, eventually aided by adhesive pastes or by means of teeth or ad hoc implants [8].

The choice criteria of treatment also depend on the dimensions of the lesion, which can be categorized using the Okay et al. classification [14].

The Okay et al. classification is based on the size and location of the defect, as well as the involvement of the denture part, complemented with subclasses when the lesion involves the zygomatic bone and the orbital floor [14].

Here, we describe three cases where the surgical management of CIMDL included the use of a temporalis muscle flap combined with a Le Fort I osteotomy, with the aim of showing how this approach indeed allows the muscle a sufficiently large space to be settled.

## 2. Case Series

### 2.1. Case 1

A 49-year-old female patient came to our attention in 2021, presenting a palatal/oro-nasal fistula measuring 2 cm in diameter along the major axis and with atrophic and burned soft tissue surrounding the lesion (Ia according to the Okay et al. classification [14]).

**Figure 1:**
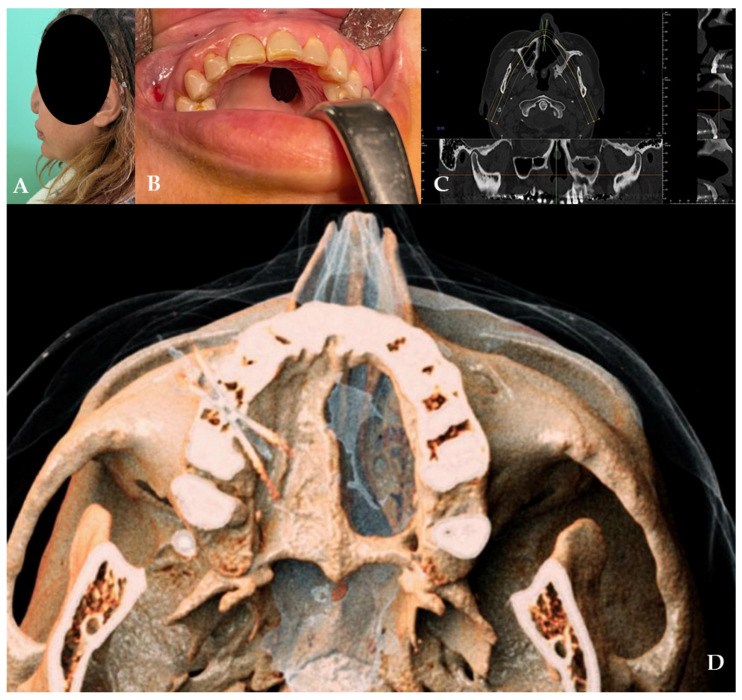
(**A**,**B**) Clinical presentation of the oro-nasal communication. In inset (**A**), it is possible to appreciate the collapse of the nasal pyramid. (**C**,**D**) Radiographic and 3D reconstruction using InVivo6^®^ and Anatomage Table EDU vers.8^®^ (Santa Clara, CA, USA) software. DICOM files were imported in InVivo6^®^ (Santa Clara, CA, USA) software to obtain the axial, cross, and panorex sections. Afterwards, the DICOM files were imported Anatomage Table EDU vers.8^®^ (Santa Clara, CA, USA) to obtain the 3D rendering.

The patient was treated with an obturator to permit correct speech and feeding. At the clinical and radiographic examination, there was also wide, bony, and cartilaginous septal destruction with communication of the nasal cavity and collapse of the nasal pyramid. Speech disturbances, rhinolalia, and eating difficulties were observed (Figure 1).

**Figure 2:**
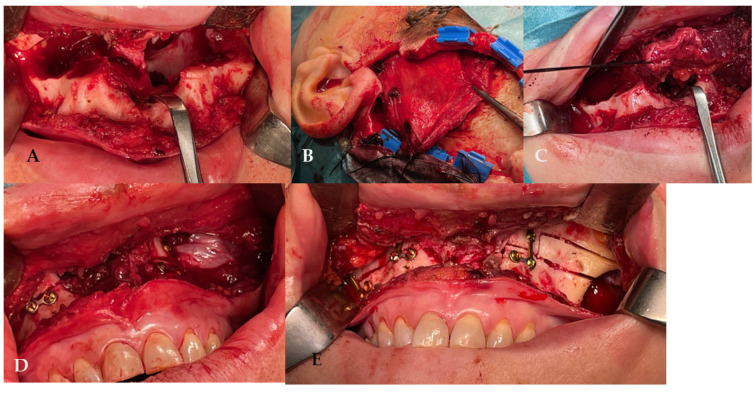
(**A**) The surgery was performed using a Le Fort I osteotomy preceded by preplating to achieve the correct repositioning of the fragment; a careful dissection of the nasal and palatal mucosa was performed after the down fracture. (**B**,**C**) Temporalis muscle harvesting and rotation (**D**,**E**) fixation of the maxillary fragments.

In 2022, after 2 years of sobriety, the patient underwent surgical intervention for palate reconstruction. Under general anesthesia, the surgery was performed, starting with a Le Fort I osteotomy preceded by preplating to achieve the correct repositioning of the fragment. A careful dissection of the nasal and palatal mucosa was performed after the down fracture. A temporalis muscle rotation flap was harvested on the left side (Figure 2). The operation lasted three hours in the oro-tracheal tube. A nasogastric tube was placed and kept in place for seven days with standard antibiotic and anti-inflammatory therapy. The patient was discharged after four days.

**Figure 3:**
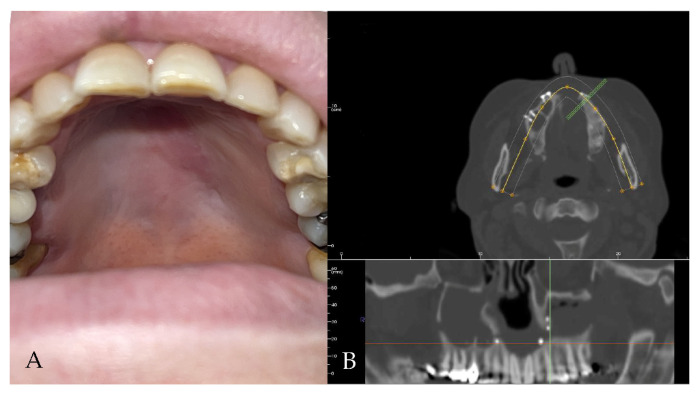
(**A**,**B**) Clinical and radiological follow-up. The palatal fistula appears closed.

The patient attended follow-up appointments at 1 week, 1 month, 6 months, and 1 year, with complete resolution of the fistula, an improved ability to speak, and no issues with eating, as confirmed by the clinical and radiographic controls (Figure 3).

### 2.2. Case 2

**Figure 4:**
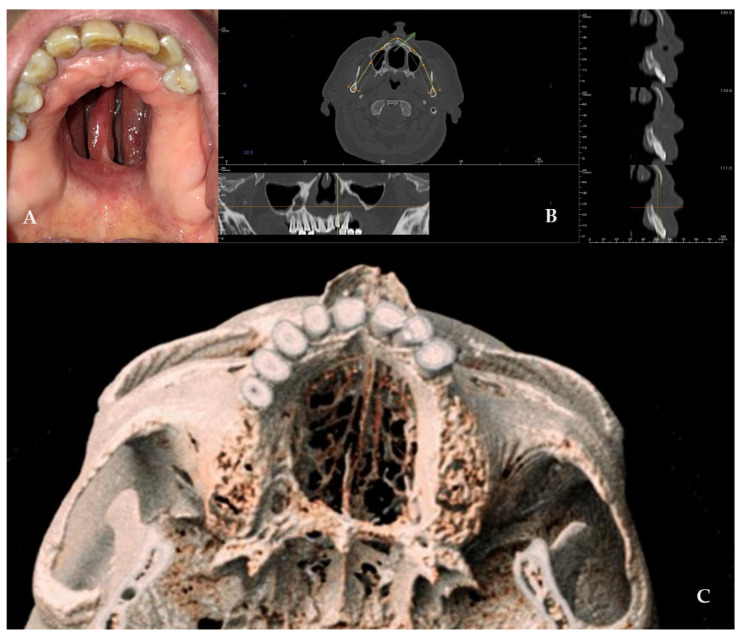
(**A**) Clinical presentation of the oro-nasal communication. In inset A, it is possible to appreciate the visibility of the nasal septum. (**B**,**C**) Radiographic and 3D reconstruction using InVivo6^®^ and Anatomage Table EDU vers.8^®^ (Santa Clara, CA, USA) software. DICOM files were imported in InVivo6^®^ (Santa Clara, CA, USA) software to obtain the axial, cross, and panorex sections. Afterwards, the DICOM files were imported into Anatomage Table EDU vers.8^®^ (Santa Clara, CA, USA) to obtain the 3D rendering.

A 52-year-old male patient came to our attention in 2017, presenting with a palatal/oro-nasal fistula measuring 3.5 cm in diameter along the major axis, with atrophic soft tissues surrounding the lesion (Ia according to the Okay et al. classification [14]). The patient had already been treated in 2015 in another center using a palatal local flap with no success and was then treated with an obturator. At the clinical and radiographic examination (Figure 4), a wide oro-nasal communication with exposure of the nasal cavity and septum was observed, leading to speech disturbances, rhinolalia, and eating difficulties.

**Figure 5:**
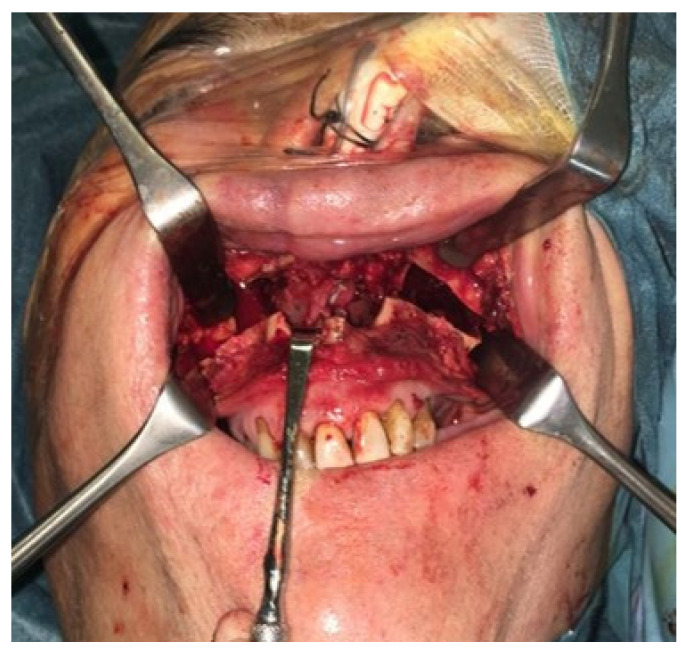
The surgery was performed, using a Le Fort I osteotomy preceded by preplating to achieve the correct repositioning of the fragment; a careful dissection of the nasal and palatal mucosa was performed after the down fracture.

In 2018, after 4 years of sobriety, the patient underwent surgical intervention for palate reconstruction. The Le Fort I osteotomy was preceded by preplating to achieve the correct repositioning of the fragment (Figure 5). A careful dissection of the nasal and palatal mucosa was performed after the down fracture. A temporalis muscle rotation flap was harvested on the right side. The operation lasted three hours in the oro-tracheal tube. A nasogastric tube was placed and kept in place for seven days with standard antibiotic and anti-inflammatory therapy. The patient was discharged after four days.

**Figure 6:**
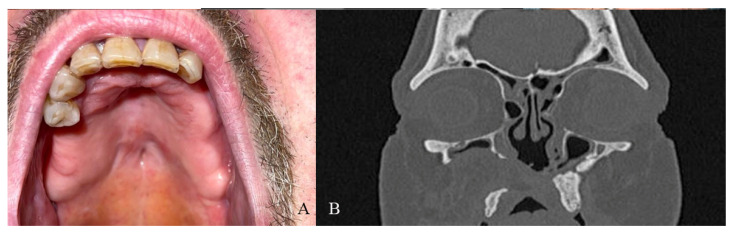
(**A**) One-year clinical follow-up. (**B**) Radiological one-year follow-up.

The patient attended follow-up appointments at 1 week, 1 month, 6 months, and one year, with complete resolution of the fistula. The patient showed an improved ability to speak and no issues with eating, as confirmed by clinical and radiographic controls (Figure 6).

### 2.3. Case 3

A 40-year-old female patient came to our attention in 2022, presenting a palatal/oro-nasal fistula measuring 2 cm in diameter along the major axis, with atrophic and burned soft tissues surrounding the lesion (Ia according to the Okay et al. classification [14]), as well as the loss of the right orbital floor and the eye. The patient was surgically treated to remove the inflamed and necrotic bone in another center, with the reconstruction of the orbital floor using a mesh. A wide bony and cartilaginous septal destruction with communication of the nasal cavity and collapse of the nasal pyramid was also shown at the clinical examination. Speech disturbances, rhinolalia, and eating difficulties were shown. In 2023, the patient underwent surgical intervention to reconstruct the palate. Under general anesthesia, the surgery was performed, starting with a Le Fort I osteotomy preceded by preplating to achieve the correct repositioning of the fragment. A careful dissection of the nasal and palatal mucosa was performed after the down fracture. A temporalis muscle rotation flap was harvested on the right side. The operation lasted three hours in the oro-tracheal tube. A nasogastric tube was placed and kept in place for seven days with standard antibiotic and anti-inflammatory therapy. The patient was discharged after four days.

**Figure 7:**
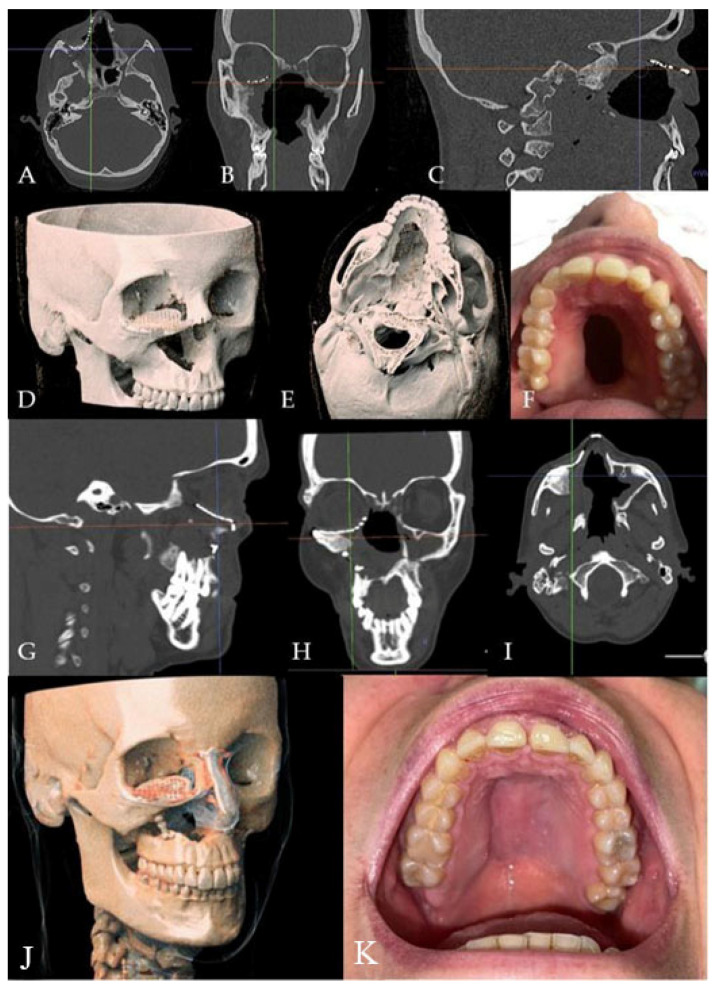
(**A**–**E**) Radiographic and 3D reconstruction using InVivo6^®^ and Anatomage Table EDU vers.8^®^ (Santa Clara, CA, USA) software. DICOM files were imported in InVivo^®^ (California, USA) software to obtain the axial, cross, and panorex sections. Afterwards, the DICOM files were imported into Anatomage Table^®^ (Santa Clara, CA, USA) to obtain the 3D rendering. (**F**) Clinical presentation of the oro-nasal communication. (**G**–**J**) Radiographic and 3D reconstructions using InVivo6^®^ and Anatomage Table EDU vers.8^®^ (Santa Clara, CA, USA) software at the one-year follow-up. (**K**) One-year clinical follow-up.

The patient attended follow-up appointments at 1 week, 1 month, 6 months, and 1 year, with complete resolution of the fistula, improved speech capacity, and no difficulties with eating (Figure 7).

## 3. Discussion

### 3.1. CIMDL Syndrome and Proposed Techniques to Face This Challenge

The surgical reconstruction of palate and nose defects due to cocaine abuse is a challenging demand for maxillofacial surgeons and ENT specialists. Indeed, the midfacial region is crucial for aesthetics and functionality. The midface, with the nose, palate, and malar bone, contributes to the appearance of facial aesthetics [15] beyond being the main components of respiratory, phonetic, digestive, and sensory systems [16].

The surgical approaches that aim to treat palate defects, such as cleft palate or post-oncological defects, employ local or regional pedicled flaps [17]. Local pedicle flaps include a tongue flap, a buccinator myomucosal flap, and a buccal fat pad graft.

The use of the tongue flap for the repair of oral structure defects was first employed in 1901 by Eiselberg [18], but the use of tongue tissues specifically for palate repair began in 1966 [19].

The choice of the tongue is due to its rich vascular supply, which ensures the creation of high-quality versatile flaps that can be raised in any direction [20].

The vascular anatomy of the tongue allows for the closure of soft or posterior hard palate defects using a posterior midline flap and the closure of anterior defects using an anterior midline flap [21,22].

For hard palate reconstructions, the tongue offers several flap options:

- Dorsal tongue flap: this flap is indicated for both anterior (hard palate, anterior buccal mucosa, anterior floor of the mouth, or lip) and posterior defects (soft palate, retromolar region, or posterior buccal mucosa) [23]. The flap should be as free from circumvallate papillae as possible [24], long enough to cover the defect, and maintain tongue functionality. After 14–21 days post-operation, the pedicle is severed.
- Lateral tongue flap: this flap design is indicated for the treatment of the buccal mucosa, the lateral palate, and oroantral communications. The design includes incisions on the ventral and dorsal surfaces of the tongue, excluding the circumvallate papillae [25].

Patients who undergo the pedicled tongue flap are prescribed a liquid diet until the pedicle separation. If necessary, mouth opening is limited by means of Barton’s bandages or fixation of the two arches [18].

Some authors suggest stabilizing the tongue to the upper lip with suture wires or fabricated aluminum splints. Post-operative complications include hemorrhages, hematoma, temporary sense loss, and difficulties in speech [18].

The buccinator myomucosal flap was first introduced by Bozola in 1989 for restoring intraoral and cleft palate defects [26]. This flap includes both pedicled and free variants, and the choice between the two depends on the arterial vascular supply [27], which is provided by the maxillary artery [26].

While raising the flap, the boundaries include the parotid duct posterosuperiorly, the oral commissure anteriorly, and the pterygomandibular raphe posteriorly [26].

Due to the importance of vascularization for the success of the surgical intervention, instruments such as Doppler ultrasound can be used to identify the route of the vessels, ensuring their inclusion in the flap [28].

The technique then proceeds with the incision of the vestibular mucosa and the buccinator muscle at the buccopharyngeal fascia level. After the flap elevation (anteriorly or posteriorly) in the loose areolar plane between the buccinator muscle and the buccopharyngeal fascia, the artery that will supply the flap is identified [27]. The dissection continues between the artery and the buccopharyngeal fascia, towards the origin of the vascular pedicle, leaving the vessel highly visible with dissecting scissors [29]. The integrity of the buccopharyngeal fascia prevents the collapse of the buccal fat and potential damage to branches of the facial nerve [28]. Negative aspects of this type of flap include tooth loss and a bite block to protect the pedicle from teeth [28].

Finally, the buccal fat pad flap is widely used for repairing small and medium-sized intraoral defects secondary to trauma and ablative resection, as well as for the closure of oroantral communications, including those due to cocaine abuse [29].

The technique includes a 2 cm mucosal horizontal incision after local anesthesia at the level of the superior second molar tooth, directed posteriorly [29].

This area hosts the buccinator muscle, whose tension is controlled by adequate lateral retraction, generally using a Minnesota retractor [29]. Once the maxillary periosteum is visible, the buccal fat pad can be found superiorly and laterally. A further incision on the maxillary periosteum, using monopolar cautery, is made along the length of the mucosal flap. This incision allows the buccal fat pad to protrude and to be isolated from the adjacent anatomical structures using Dean scissors [29].

The use of DeBakey forceps for actively pulling the fat pad is not advisable since it could damage vascular integrity. However, DeBakey forceps can be used to restrain the flap once adequate tissues are isolated and then excised [29].

The buccal fat pad is then sutured tightly to close the defect, without needing mucosal covering. Indeed, re-epithelization occurs naturally by the fourth post-operative week [30]. Regional flaps include the temporalis muscle flap.

Regarding the use of free flaps that are microvascularized in the region, these are indicated in medium and large defects.

Free flaps provide available tissue with adequate blood supply, which can cover the defects without tension [30].

Possible options are the anterolateral thigh, the brachial, the parascapular, and the free forearm fasciocutaneous vascularized tissue [9]. Colletti et al. highlighted the advantages of the forearm free flap, as it appears versatile and thin, has a long vascular pedicle, and is ideal for facial vessel anastomosis [31]. The chronic abuse of cocaine causes an inflammatory process throughout the body. Even though Colletti et al. underlined the success of free flaps [31], damage to facial vessels might compromise the long-term stability of the forearm flap. Pedicled local and regional flaps keep the vascular supply intact without needing microvascularization or other anastomoses.

Alternatively, an obturating prosthesis is a good solution in cases of surgical failure or before recovery from addiction to the drug [32]. The obturator is adjusted to the dimensions of the defect, ensuring complete sealing to the maxillary teeth and facilitating correct oral functions [8]. The manufacturing of prosthetic obturators includes a metal framework and an acrylic resin structure, covering the fistula and eventually providing a bulb or a pharyngeal extension [8].

### 3.2. Temporalis Flap and Le Fort I Modifications

The temporalis muscle flap is generally considered a durable flap with a very low incidence of failure (1.6%) because it has a reliable and predictable vascular pedicle [33,34,35]. Indeed, the temporalis muscle is characterized by high flexibility and a large rotational arc (approximately 135°), which make it ideal for the defects of the temporal bone, anterior skull base, midfacial bone structures (hard palate, soft palate, orbital cavity, and maxillary bones), and oral cavity [36].

The first incision starts from the preauricular crease and extends superiorly, with a hemicoronal shape, following the line of the temporal fossa. The incision can be placed behind the hairline to minimize unsightly depressions. After anesthesia, the dissection starts through the skin, passing the subcutaneous layer and temporoparietal fascia. The skin flap, including the temporoparietal fascia, is then elevated from the root of the zygomatic process to the lateral orbital wall. Finally, the deep temporal fascia is reached. The superficial layer of the deep temporal fascia is incised and dissected within the superficial temporal fat pad to guarantee the protection of the facial nerve. The superficial temporal fat pad can be divided using a hemostat to protect the underlying muscle. Afterward, the zygomatic arch is exposed, with the retraction of the periosteum, which is crucial for the protection of the frontotemporal branches of the facial nerve. If the entire muscle is necessary for the reconstruction, the cutaneous part is separated from the muscle, which, once free from the attachment and insertions, can be rotated and passed through the zygomatic arch to reach the midfacial or oral cavity structure under repair [36].

The correct orientation of the muscle is guaranteed by a strong suture on the deep temporalis fascia. The flap is then sutured on the defects, orienting the fascia to the oral cavity. Finally, the donor site is closed with drainage and a pressure dressing [36].

If the arc of rotation must be increased, the zygomatic arch can be modified by means of osteotomies and repaired with titanium at the end of the procedure. The coronoid process can also be cut to allow muscle rotation, maintaining the blood supply [36]. If only a minor portion of the muscle is necessary, the muscle can be coronally sectioned using only the anterior part [36].

When the passage is not large enough, it can strangle the muscle, causing ischemia and necrosis of the flap [37].

The proposed solution presents its innovation in the Le Fort I osteotomy, which avoids muscle strangulation and guarantees adequate space for placing the temporalis flap, preserving the muscle and the blood supply.

In addition, tissue loss due to cocaine abuse results in an altered anatomy. The Le Fort I osteotomy, although it might appear to be an additional and even invasive surgical technique, helped in all three cases in achieving a cleaner surgical procedure, with a clear vision of the altered anatomy. Regarding the management of the temporal muscle, the addition of the Le Fort 1 osteotomy prevents flap strangulation and maintains the integrity of the vascular pedicle.

Bone grafting was not chosen as cocaine abuse significantly alters the local vascularization [12], as seen in other clinical situations where the receiving site has a damaged local vascular supply [13]. Therefore, a vascular axis offers a higher success rate.

Finally, the presented cases did not present any challenges during the surgical procedures. 

An accurate pre-surgical study of the lesion is mandatory. Indeed, ischemic lesions can sensibly modify the anatomy and the margins of the fistula, and a radiologic volumetric assessment greatly contributes to planning the surgical steps and avoiding and managing any hemorrhagic and neurological complications. As with every surgical procedure, the reported one in this study also requires an adequate learning curve before it can be performed.

The study of the lesion on the 3D renderings, as well as on the radiographs, was crucial for planning the surgical approach.

Within the limits of this study, including its retrospective nature and the small sample size, we can affirm that despite the diversity of the patients’ lesions, an accurate pre-surgical study, which included the medical and radiological evaluations, surgical steps, and a clear vision of the surgical field, simplified the management of the temporal muscle flap.

### 3.3. Influence of Cocaine on the Choice of CIMDL Rehabilitation Treatment 

The described hard and soft palate closure techniques are valid and reliable, and each can be used according to the dimensions of the defects. However, the use of free flaps has recently become very popular due to the possibility of reconstructing wide defects with better vascularization and without tension [31].

Nonetheless, chronic cocaine abuse repeatedly and constantly damages the vessels’ endothelium, not only locally in the midfacial structures but throughout the whole body.

For example, the heart suffers at the ventricular level, with a decrease in the ejection fraction and increased end systole due to the interaction between cocaine and norepinephrine [12].

In addition, reports in the literature showed how chronic cocaine use impacts the nervous system, with panhypopituitarism, dysfunction of the optic nerve, diffuse lesions in white matter, including the basal ganglia, proptosis, and visual dysfunction due to the destruction of the bone components of the orbital cavity [12].

In general, cocaine triggers the immune system and induces an inflammatory process, decreasing anti-inflammatory markers such as interleukin-10 and increasing pro-inflammatory cytokines (e.g., tumor necrosis factor alpha and interleukin 1β). The activation of this cytokine cascade contributes to vascular disease [12].

All of these considerations, combined with the addiction disturbance, make CIMDL rehabilitation a challenge.

### 3.4. The Le Fort I Osteotomy: Is It Always Necessary?

The reported cases showed how cocaine consumption can lead to a serious pattern of lesions involving the maxilla and the nose bone–cartilage complex.

Therefore, it appeared that an adjunctive but established surgical step such as the Le Fort I Osteotomy could offer a wider view of the surgical field. The temporalis flap indeed includes a partial osteotomy of the maxilla. However, the bone cut offered by the Le Fort I osteotomy in the reported cases provided a clear vision of the surgical field and of the altered anatomy of the cocaine-induced lesions.

## 4. Conclusions

CIMDL represents a diagnostic and treatment rehabilitation challenge for ENT and maxillofacial surgeons. Among all of the available options, using a Le Fort I osteotomy and a temporalis muscle flap might represent an efficient technique for correcting medium- and large-sized oro-nasal communications that are unresolvable with simple oral mucosa flaps or obturators. When performed with only the anterior part, there may be a minimal impact on the patient’s aesthetic appearance. The pedicled flap, along with the presented modification, represents a therapeutic option to be considered in patients whose endothelial tissue is damaged by chronic cocaine abuse.

## Data Availability

Data will be available from the corresponding author upon kind request.
